# Sushi.R: flexible, quantitative and integrative genomic visualizations for publication-quality multi-panel figures

**DOI:** 10.1093/bioinformatics/btu379

**Published:** 2014-06-05

**Authors:** Douglas H. Phanstiel, Alan P. Boyle, Carlos L. Araya, Michael P. Snyder

**Affiliations:** Department of Genetics, Stanford University School of Medicine, Stanford, CA 94305, USA

## Abstract

**Motivation:** Interpretation and communication of genomic data require flexible and quantitative tools to analyze and visualize diverse data types, and yet, a comprehensive tool to display all common genomic data types in publication quality figures does not exist to date. To address this shortcoming, we present Sushi.R, an R/Bioconductor package that allows flexible integration of genomic visualizations into highly customizable, publication-ready, multi-panel figures from common genomic data formats including Browser Extensible Data (BED), bedGraph and Browser Extensible Data Paired-End (BEDPE). Sushi.R is open source and made publicly available through GitHub (https://github.com/dphansti/Sushi) and Bioconductor (http://bioconductor.org/packages/release/bioc/html/Sushi.html).

**Contact:** mpsnyder@stanford.edu or dphansti@stanford.edu

## 1 INTRODUCTION

Genomic science is a rich data-intensive field in which diverse data types are combined to uncover and explore characteristics of sequence elements on a large scale. However, despite a growing set of mature standard visualization techniques and file formats, no comprehensive tools exist to facilitate multi-panel visualization across a broad range of standard genomic data types. To address this deficiency, we developed Sushi.R, a flexible R library that leverages standard visualization techniques and file formats to produce highly customizable publication-quality figures of genomic data within the widespread analysis environment, R(R Core Team, 2013).

## 2 METHODS

**Fig. 1.**
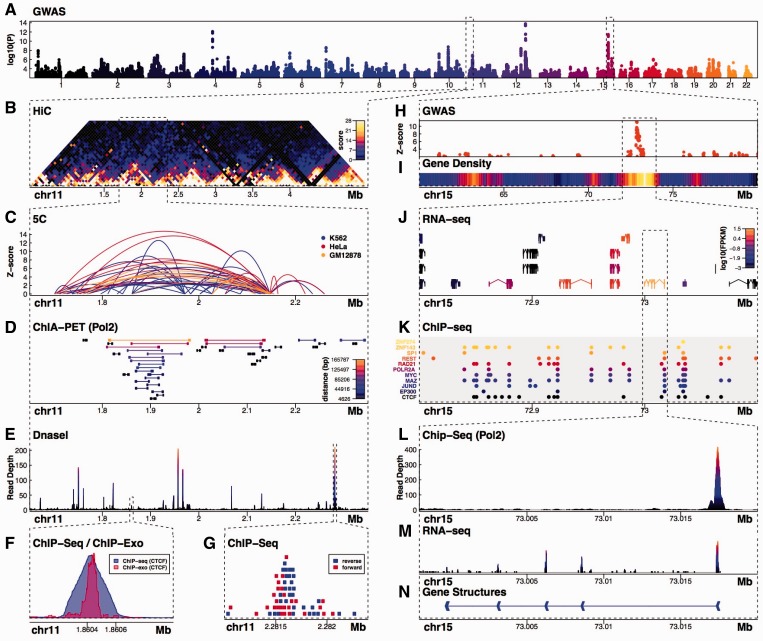
Multi-panel Sushi plot made without modification by external image-editing software. The Sushi functions used to create the plot include (**A**) *plotManhattan*, (**B**) *plotHic*, (**C**) *plotBedpe*, (**D**) *plotBedpe*, (**E**) *plotBedgraph*, (**F**) *plotBedgraph*, (**G**) *plotBed*, (**H**) *plotManhattan*, (**I**) *plotBed*, (**J**) *plotGenes*, (**K**) *plotBed*, (**L**) *plotBedgraph*, (**M**) *plotBedgraph* and (**N**) *plotGenes*. The code and data to make this figure are included as part of the Sushi.R package

Sushi.R is written exclusively in the R software environment. The Sushi.R package includes 13 example datasets and a vignette detailing the usage of each (Sanyal *et al.*, 2012; Li *et al.*, 2012; ENCODE Project Consortium *et al.*, 2012; Neph *et al.*, 2012; International Consortium for Blood Pressure Genome-Wide Association Studies *et al.*, 2011; Dixon *et al.*, 2012; Rhee and Pugh, 2011). Datasets that were mapped to hg19 were converted to hg18 using the liftOver tool. Sushi is compatible with all organisms and genome builds. Large datasets were filtered to include only regions shown in Figure 1. ChIA-PET interactions were additionally filtered to remove interactions between regions ≤1000 bp apart. To facilitate use, Sushi.R is open source and is distributed through both Bioconductor for one-step installation and GitHub for version control, issue management and third-party development (Gentleman *et al.*, 2004).

## 3 FEATURES

Quantitative and qualitative genomic information can typically be broken down into three data types: features, signals and interactions. Sushi.R provides flexible methods to plot each data type, allowing users to represent virtually any type of genomic data in an aesthetically pleasing, coherent and integrative fashion. A Sushi plot made entirely within R (without any modifications in image-editing software) displaying multiple data types is shown in Figure 1. The code and data to make Figure 1 are included as part of the Sushi.R package.

Feature data describe genomic regions characterized by a unique combination of chromosome, start and stop coordinates. Often stored in Browser Extensible Data (BED) format, feature data can be used to represent sites of transcription factor binding, gene structures, transcript structures, sequence read alignments, Genome-Wide Association Studies (GWAS) hits and data from an array of other sources. The Sushi functions *plotBed*, *plotGenes* and *plotManhattan* facilitate the visualization of feature data in a host of different formats ranging from heatmaps of feature density to feature pileups (Figure 1A, G–K, N).

Signal data representing quantitative values across genomic coordinates are commonly stored in bedGraph format and can be used to represent diverse forms of data including sequence conservation, transcription, transcription factor binding, chromatin accessibility and nascent transcription rates, among others. The Sushi function *plotBedgraph* provides flexible methods to plot, overlay and compare signal track data with appropriately represented data from each one of these disparate sources (Figure 1E, F, L, M).

Finally, interaction data can be used to describe interactions between distal genomic elements in both a qualitative or quantitative fashion. Interaction data describing, for example, 3D chromatin structure are commonly stored in Browser Extensible Data Paired-End (BEDPE) format or in interaction matrices. Sushi functions *plotHiC* and *plotBedpe* are used to plot interactions data as either trapezoidal heatmaps, arched lines or box and line structures, and support quantitative mapping of interaction signals on *y*-axis values, color scales and line widths (Figure 1B–D).

Sushi plots can easily be combined and augmented *via* a number of annotation functions including *zoomsregion*, *zoombox*, *maptocolor* and *addlegend*, allowing customizable scaling of colors, line types and line widths for flexible quantitative presentation. Zoom inset features facilitate visualization at multiple scales and diverse genomic contexts. Images can be written to all formats supported by R including Encapsulated PostScript (EPS), Portable Document Format (PDF) and Portable Network Graphics (PNG).

## 4 DISCUSSION

The rapid proliferation and complexity of genomics experiments—fueled by high-throughput sequencing—has concomitantly driven demand for analysis and visualization tools that facilitate interpretation and communication of rich and diverse genomic data types. Sushi fills a critical void among currently available visualization tools by providing a means to easily produce sophisticated, customizable, genomic visualizations. Sushi.R will be of great use to the genomic community, as it accelerates our ability to uncover, document and communicate important scientific findings derived from increasingly abundant, and complex, genomic data.

*Funding*: This project is funded by NIH grant U54HG006996 (to M.P.S) and K99HG007356 (to A.P.B). D.H.P. is a Damon Runyon fellow supported by the Damon Runyon Cancer Research Foundation (DRG-2122-12).

*Conflict of interest:* M.P.S. is a cofounder and scientific advisory board (SAB) member of Personalis and also on the SAB of Genapsys.
